# Vanadium-Based MXenes: Types, Synthesis, and Recent Advances in Supercapacitor Applications

**DOI:** 10.3390/nano15131038

**Published:** 2025-07-04

**Authors:** Zhiwei Gao, Donghu Shi, Jiawei Xu, Te Hai, Yao Zhao, Meng Qin, Jian Li

**Affiliations:** 1 Hubei Key Laboratory of Energy Storage and Power Battery, School of Optoelectronic Engineering, School of New Energy, Hubei University of Automotive Technology, Shiyan 442002, China; 202310070@huat.edu.cn (Z.G.); 202410073@huat.edu.cn (D.S.); 202311155@huat.edu.cn (J.X.); 202311153@huat.edu.cn (T.H.); 202310072@huat.edu.cn (Y.Z.); 2 Hubei Key Laboratory of Energy Storage and Power Battery, School of Automotive Materials, Hubei University of Automotive Technology, Shiyan 442002, China

**Keywords:** two-dimensional material, V_2_CT*_x_*, V_4_C_3_T*_x_*, derivatives, supercapacitor applications

## Abstract

Since the discovery of two-dimensional transition metal carbides and nitrides (MXenes), MXenes have attracted widespread research in the academic community due to their advantages, such as adjustable interlayer spacing, excellent hydrophilicity, conductivity, compositional diversity, and rich surface chemical composition. More than 100 different MXene combinations can be calculated theoretically, but only more than 40 have been successfully synthesized through experiments. Among the many synthesized and reported MXene materials, vanadium-based carbide MXenes, represented by V_2_CT*_x_* and V_4_C_3_T*_x_*, show excellent application prospects in energy storage and have become the focus of researchers. In this review, we mainly discuss the structure, characteristics, and preparation methods of vanadium-based MXene precursors in the MAX phase and their applications in supercapacitors. Finally, we propose the main challenges existing at the current stage of vanadium-based materials and their heterostructures and provide a perspective on future research directions.

## 1. Introduction

In addressing global energy transition and the challenges of renewable energy grid integration, developing new electrochemical energy storage materials that combine high power density and high energy density has become a vital frontier topic in energy science [1,2,3]. As a core component of the electrochemical energy storage system, supercapacitors, with their ultra-fast charge transfer kinetics and cycle life, hold an essential position in transient power supply scenarios such as smart grid peak shaving and electromagnetic catapult systems [4,5]. However, due to the limitation of the double-layer physical adsorption energy storage mechanism, there is an order of magnitude difference in their intrinsic energy density compared to battery systems. This key performance shortcoming seriously constrains their application prospects in large-scale energy storage fields [6].

**Figure 1:**
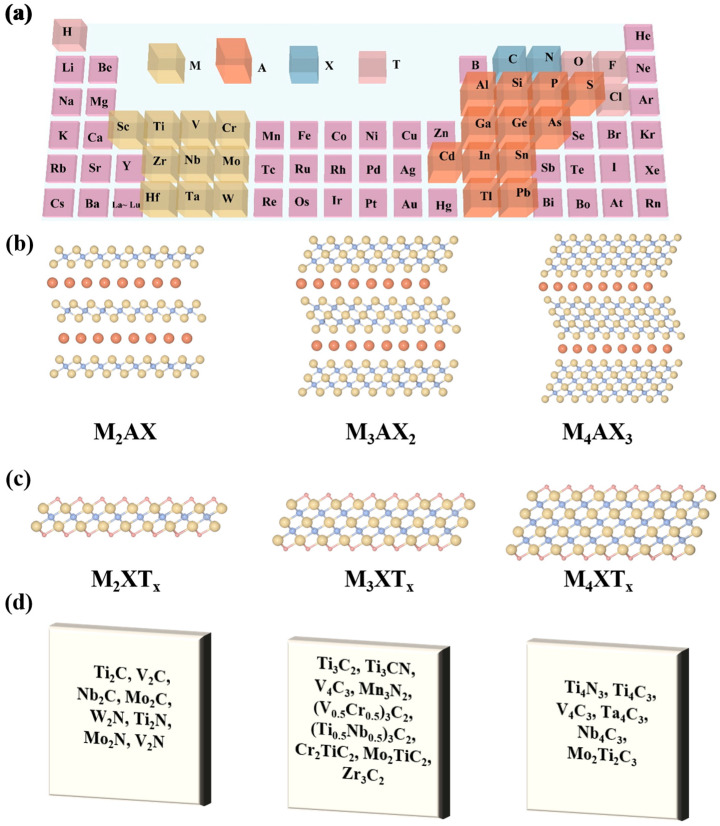
(**a**) Fragment of the periodic table of elements, the “M”, “A”, “X” and “T” elements in MAX phases. (**b**) The structure of a MAX phase and (**c**) the corresponding MXA. (**d**) A typical MXene.

The breakthrough progress in two-dimensional nanomaterials has provided a new theoretical framework for the design of energy storage materials. Following the successful synthesis of the two-dimensional carbonaceous material graphene [7], two-dimensional material systems represented by transition metal chalcogenides (such as MoS_2_, WS_2_) [8,9,10] and layered double hydroxides (such as NiCo-LDH) [11,12], due to their unique quantum confinement effect and surface pseudocapacitance characteristics, have sparked continuous research enthusiasm in the field of energy storage [13,14]. Notably, MXenes (transition metal carbides/nitrides), as new members of the two-dimensional material family, since their successful preparation in 2011 through hydrofluoric acid etching of MAX phase precursors [15], have achieved significant progress in structural regulation strategies and energy storage application research [16,17,18]. The synthesis of these materials follows specific topological chemical rules: by selectively removing the weakly bonded “A” atom layer (typically Al, Si elements) from the MAX phase, M*_n_*_+1_X*_n_*T*_x_* structures (*n* = 1–3) with alternating stacking are obtained, where M represents transition metals like Ti and V, X is C or N atoms, and T*_x_* denotes surface terminating functional groups (–O, –OH, –F, etc.) [19,20,21]. As shown in Figure 1a,b, the unique metal-like conductivity and large interlayer spacing of MXenes make them possess rapid ion transport capabilities and a high Faraday pseudocapacitance contribution rate, becoming an ideal electrode material system for constructing the next generation of high-performance supercapacitors [22,23,24,25,26,27].

MAX phase materials have a typical hexagonal close-packed crystal structure, and their unit cells consist of alternating stacked MX hexagonal layers and A atomic layers [28,29,30]. Depending on the difference in the value of *n* in the chemical formula (*n* = 1, 2, 3), the layered structures of derived MXenes (M*_n_*_+1_X*_n_*T*_x_*) can present different stacking configurations such as 3, 5, and 7 layers [31,32,33,34,35]. Theoretical predictions show over a hundred possible combinations in the MXenes family, but only 40 have been synthesized experimentally (Figure 1d). Based on atomic arrangement features, MXenes can be divided into two major structural types: a solid solution phase (random distribution of multiple metals) and an ordered phase (ordered interlayer of transition metals) [36,37]. Among them, M_2_X-type MXenes can be classified into single-metal and bimetallic systems according to the composition of the metal layer. The increase in the number of metal layers will cause a compression effect on the interlayer spacing, and this structural evolution will directly affect the ion diffusion barrier, the number of electrochemical active sites, and the mechanical properties of the material [38]. By precisely controlling the surface chemical composition and interlayer spacing of MXenes, the conductivity, mechanical strength, and electrochemical activity performance parameters can be effectively optimized.

Current research mainly focuses on the Ti_3_AlC_2_-phase-derived Ti-based MXene system, while vanadium-based MXenes (V-MXenes) possess unique redox reversibility (V^2+^/V^3+^/V^4+^) and ultra-high theoretical specific capacity. However, they face technical bottlenecks such as controllable etching and efficient exfoliation due to the difficulty in regulating surface functional groups and strong interlayer coupling [39]. Among the reported vanadium-based MXenes, V_2_CT*_x_* and V_4_C_3_T*_x_* have become a hot topic in energy storage research due to their distinct interlayer channel characteristics. In response to the challenges in their preparation techniques, the academic community has proposed a series of synthesis strategies, including in situ etching with hydrofluoric acid, fluoride salt-assisted etching (such as NaF/LiF system), and electrochemical gradient etching [40]. It is worth emphasizing that the multivalent nature of vanadium endows V-MXenes with excellent charge storage kinetics. By compositing with conductive polymers or transition metal oxides to form heterogeneous interfaces, the composite materials’ specific capacitance and cyclic stability can be significantly improved. Simultaneously, metal–organic framework materials (MOFs), carbon nanofibers (CNFs), and layered double hydroxides can also be supercapacitor materials. V-based MXenes and MOFs show significant differences in structure and performance. V-based MXenes, as a two-dimensional transition metal carbide, are obtained by etching the MAX phase with hydrofluoric acid and have conductivity like metals and tunable interlayer spacing. Still, they have a lower specific surface area and are easily oxidized. On the other hand, MOFs are three-dimensional porous crystals constructed from metal clusters and organic ligands. They have a higher specific surface area and porosity but inherently poor conductivity [41]. Therefore, due to their high conductivity, V-based MXenes perform prominently in supercapacitors and zinc-ion battery electrode modifications. At the same time, MOFs are more advantageous in gas storage and catalysis due to their designable pore channels. Using the electrospinning method, CNFs can be massively produced with a porous/hollow structure. Typically, V-based MXene is combined with CNFs to utilize its porous structure to buffer volume expansion, resulting in long-term stability characteristics in supercapacitors [42]. Its high flexibility can enhance device bendability, expanding its application field. Layered double hydroxides (LDHs) generally comprise divalent or trivalent metal layered hydroxide nanosheets with interchangeable anions between layers, maintaining interlayer equilibrium through electrostatic interactions. Using V-based MXene as a conductive substrate to form a composite can accelerate the charge transfer kinetics of LDHs and reduce the overpotential of the oxygen evolution reaction (OER), and the synergistic active sites between the LDH nanosheets can provide an efficient catalytic center, enhancing the catalytic reaction efficiency [43]. Moreover, the ultra-thin two-dimensional layered structure of V-based MXene can significantly inhibit the agglomeration of LDHs, increasing its specific surface area. The interlayer confinement effect of LDHs can also slow the oxidation of MXenes by anchoring –OH/–O functional groups. Therefore, constructing a heterostructure combining V-based MXene with LDHs can synergistically leverage both advantages, showing immense industrial application potential in supercapacitors.

The specific challenges of the HF-free synthesis route for V-MXene are as follows: Firstly, vanadium-based MXenes are generally prepared from the precursor V_2_AlC to produce V_2_C. The bond energy of V-Al is significantly higher than that of Ti-Al, making it difficult for traditional HF-free etchants to break effectively, leading to residual Al layers [25]. Secondly, vanadium elements are easily oxidized to higher valence states (V^4+^/V^5+^), especially in oxygen-containing environments, forming inactive oxides (such as V_2_O_5_), and the terminal functional groups of V-based MXenes prepared by the HF-free method (T*_x_* = Cl/Br) are unstable in air. Finally, the etched accordion-structured V-based MXene generally requires the use of intercalants (TBAOH/TMAOH) to exfoliate and obtain single or few-layer MXenes [24,25,26], thereby increasing their exposed active sites and promoting their significant electrochemical performance.

This review systematically analyzes the preparation of the chemical system of vanadium-based MXenes, focusing on the reaction mechanisms and process characteristics of core synthesis technologies such as hydrofluoric acid etching, fluoride salt-assisted etching, and electrochemical etching. It deeply explores the optimization mechanisms of its electrochemical performance by strategies such as interlayer distance regulation engineering, surface termination functional group regulation, and heterostructure construction. A comprehensive review of the latest research progress of V-MXenes in supercapacitors reveals their unique advantages in high-power energy storage devices. Finally, it also looks forward to the potential research directions of V-MXenes in precise and controllable synthesis and multi-scale structure regulation.

## 2. Basic Properties of V-MXene

V-based MXenes possess multivalent characteristics, excellent conductivity, large specific surface area, adjustable interlayer spacing, and good chemical stability, endowing them with broad application potential in energy storage. Through rational design and functionalization of V-based MXenes, these materials can play a significant role in electrode materials, energy storage devices, and other fields, especially demonstrating outstanding electrochemical performance in battery and supercapacitor applications due to their unique structural advantages.

### 2.1. Multi-Valence Characteristics

The unique advantage of vanadium-based MXenes stems from the multivalent nature (+2 to +4) of the vanadium element, which enables a multi-electron transfer mechanism during electrochemical energy storage, significantly enhancing charge storage capacity [44]. In alkali metal-ion batteries and supercapacitors, the reversible redox reactions of vanadium effectively enhance the energy density and cyclic stability of electrode materials through synergistic transitions between different valence states. This characteristic is universal in vanadium-based compound systems; typical representatives include layered vanadium oxides, two-dimensional chalcogenides, and polyanion-type compounds. Their diverse crystal structures provide abundant active sites for ion storage [45].

As essential members of the vanadium-based material system, V_2_C and V_4_C_3_, with their unique two-dimensional layered structure and the multivalent characteristics of vanadium elements (reversibility of V^2+^/V^3+^/V^4+^ redox), have shown significant advantages in the field of energy storage and catalysis [28]. Their structural features are the following: the V-C layers obtained by selective etching of MAX phases (such as V_2_AlC) are stacked by van der Waals forces, with surface modifications of –O, –OH, –F, etc., terminating groups, and interlayer stacking can be suppressed through intercalation engineering or heterogeneous compounding to optimize ion diffusion dynamics, while the multivalent state characteristics of vanadium provide high-density pseudocapacitive active sites through reversible oxidation reactions (such as V^3+^↔V^4+^), endowing the material with high specific capacity and catalytic activity [37,38]. In addition, the synergistic effect of structure and valence state (such as surface amination enhancing potassium ion adsorption, heterogeneous interface accelerating charge transfer) will further strengthen its performance advantages. Still, it is necessary to overcome the bottlenecks of poor oxidation stability and dependence on hydrofluoric acid during preparation [44,45].

Vanadium trioxide (V_2_O_3_) exhibits a unique metal–insulator phase transition behavior. This material has a rhombohedral crystal structure, and its electronic properties undergo significant changes with temperature: it exhibits quasi-metallic conductivity at high temperatures. It transforms into an insulating state at low temperatures, accompanied by antiferromagnetic order. This phase transition characteristic is closely related to the special electron configuration of vanadium ions, making it of great application value in intelligent thermal control devices and phase change memory. Meanwhile, the open framework structure of V_2_O_3_ is conducive to ion implantation/extraction dynamics, combined with its intrinsic high conductivity, making it an ideal candidate for lithium/sodium ion battery anode materials [46]. It is worth noting that this material also performs prominently in catalysis, especially in the purification of nitrogen oxides and organic synthesis reactions, highlighting its multifunctional characteristics in the energy-environment intersection. Vanadium monoxide (VO), as a typical metallic conductive oxide, has the +2 oxidation state of vanadium in its cubic crystal system, endowing the material with unique electronic structure characteristics. The material has both quasi-metallic conductivity and excellent thermal stability [47]. Its chemical characteristics show a significant reductive tendency: stepwise oxidation reactions can occur in an oxidizing environment to form high-valent oxides, react with acidic media to form vanadium salt compounds, and maintain structural stability under alkaline conditions. This special chemical activity gives it significant application potential in high-temperature electrode materials and functional coatings.

Vanadium sulfide (V_2_S_3_) exhibits differentiated structural features and functional advantages in the vanadium-based sulfide system. In the three-dimensional framework of V_2_S_3_, vanadium atoms and sulfur ligands form an octahedral coordination configuration; its semiconducting properties and abundant redox active sites provide an ideal reaction interface for electrochemical energy storage. In contrast, the layered monoclinic system of vanadium sulfide promotes rapid ion diffusion through expanded sulfur–vanadium–sulfur interlayers; its intrinsic magnetism and high conductivity synergistically enhance the charge transfer rate [48]. The outstanding performance of these two types of sulfides in alkali metal ion storage systems stems from the synergistic effect of the high polarizability of chalcogen elements and weak van der Waals forces between layers, which not only ensures cyclic stability but also displays unique advantages in hydrogen evolution reactions (HERs) and oxygen reduction reactions (ORRs) [49].

### 2.2. High Surface Area and Conductivity

The preparation of vanadium-based MXenes follows the principle of topological chemical etching, which is achieved by selectively removing the Al atom layer from the ternary layered MAX phase (such as V_2_AlC, V_4_AlC_3_). The resulting two-dimensional material comprises a V-X (X = C/N) covalent bond network, with surface modifications of –OH, –O, –F, and other termination groups. The layers are held together by weak van der Waals forces, forming a peelable structure [40]. This unique chemical bonding method endows the material with dual characteristics: covalent and metallic bonds ensure structural stability. At the same time, the adjustable interlayer distance (through intercalation engineering) provides diffusion channels for ion transport [50].

Through intercalation engineering strategies (such as dimethyl sulfoxide, tetrabutylammonium hydroxide solution, or KCl intercalation), the interlayer spacing can be controlled and the interlayer interaction weakened. Combined with subsequent liquid phase exfoliation, the controlled preparation of mono/few-layer MXene nanosheets can be achieved [51]. After exfoliation, the specific surface area of the material significantly increases, exposing numerous active edge sites and interlayer storage space, effectively promoting the reversible embedding/desorption of ions such as Li^+^ and Na^+^. Notably, the electronic structure of MXenes has both metallic and semiconductor characteristics: The strong V-C/N covalent bond forms a highly conductive substrate. At the same time, the surface terminal groups regulate the interfacial electrochemical activity through charge redistribution. In energy storage applications, this structural feature gives the material metal-like conductivity and structural stability. The expanded interlayer channels significantly reduce the ion diffusion barrier, demonstrating excellent rate performance and cyclic stability in fast energy storage devices such as supercapacitors.

### 2.3. Surface Functionalization and Heterostructures

The surface termination group (–OH, –F, –O) engineering of vanadium-based MXenes is the core strategy for its functional modification. By directionally modifying the surface chemical environment through liquid phase intercalation or gas phase post-treatment technology, the interface compatibility between the material and the electrolyte can be significantly optimized: oxygen-containing groups enhance hydrophilicity to promote the infiltration of aqueous electrolytes. At the same time, fluorination treatment improves the structural stability under organic systems. This surface chemical regulation reduces the ion migration barrier (the diffusion coefficient increases by two to three orders of magnitude). It activates the pseudocapacitive behavior of edge sites by inducing local charge redistribution.

The heterostructure design further expands the performance boundaries of MXenes: The composites with conductive polymers (such as 3,4-ethylenedioxythiophene) can construct a three-dimensional ion/electron dual continuous transport network [52]; coupling with transition metal oxides (such as MnO_2_) achieves a synergistic effect of double-layer storage and Faraday reaction [53]. This structure–function integrated design endows it with the advantages of high conductivity substrate and adjustable active sites, showing a triple-gain effect in the field of energy storage: the porous interlayer channels accelerate the diffusion kinetics of multivalent ions (Zn^2+^, Al^3+^); the multivalent state transition of vanadium (V^2+^/V^3+^/V^4+^) provides high-density redox active sites; and the surface terminal groups and heterogeneous interfaces synergistically regulate the charge storage mechanism [54].

The integration of such characteristics makes V-MXenes an ideal platform for innovative energy storage devices: achieving high reversible capacity in zinc-ion capacitors, suppressing sulfide shuttle in lithium–sulfur battery systems, and possessing bending cycle stability in the field of flexible supercapacitors. Through cross-scale structural engineering and precise control of surface interfaces, this material system demonstrates the value of innovation from basic research to industrial applications.

## 3. Synthesis Methods of V-MXene

Many studies have been published on the synthesis of MXene, mainly based on the layered ternary MAX phase, using chemical etching techniques to remove the A elements, thus forming a layered MXene structure. Subsequent intercalation and exfoliation processes result in few-layer or monolayer MXene sheets with more active sites. The preparation method of V-MXene is through a top-down synthesis approach. Generally, V_2_AlC is used as a precursor, and an accordion structure is obtained by etching out the “Al” layer. The scalability of V-based MXene in industrial applications has achieved significant breakthroughs in recent years. Its high conductivity and theoretical capacity demonstrate considerable potential in energy storage. However, the traditional hydrofluoric acid etching method is limited for large-scale applications due to its high toxicity and the product’s oxidization tendency. Solid solution design and cation intercalation modification techniques have significantly improved the material’s cyclic stability and environmental adaptability [53]. For instance, interlayer ion regulation and heterogeneous interface optimization can significantly extend the lifespan of devices and enhance their low-temperature performance. Moreover, the current development bottlenecks focus on developing green synthesis processes (such as fluoride-free etching agents) and cost control of precursor raw materials. In the future, it is necessary to combine emerging technologies such as artificial intelligence to optimize material structural parameters and collectively promote industrialization in areas like flexible energy storage and wearable devices. The primary etching methods currently include hydrofluoric acid (HF), mixed-acid, hydrothermal, and other methods. In this section, commonly used etching methods are discussed.

### 3.1. Mixed-Acid Etching

**Figure 2:**
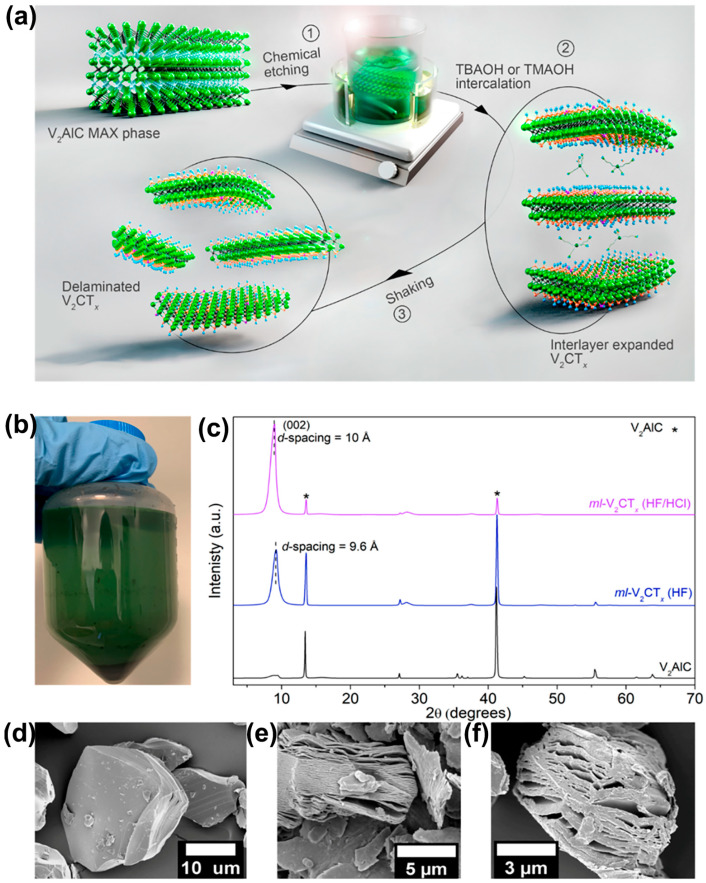
(**a**) Schematic of the MXene synthesis process. (**b**) Optical image of the supernatant after the first washing. (**c**) XRD of the MAX phase and multilayer V_2_CT*_x_* with different etching solutions. (**d**) V_2_AlC MAX phase. (**e**) HF-etched multilayer V_2_CT*_x_*. (**f**) Mixed-acid etched multilayer V_2_CT*_x_* SEM images [55]. Copyright 2021, ACS Publications.

The direct etching method with hydrofluoric acid has defects such as incomplete layered dissolution and insufficient exposure of active sites when preparing V-based MXene, which significantly restricts the improvement of its energy storage performance. Systematic research by Matthews et al. [55] shows that using a mixture of HF/HCl can dramatically improve the interlayer stripping effect compared to the traditional hydrofluoric acid system. This conclusion is verified through characterization methods such as XRD and SEM (Figure 2a). The etching process uses 12 mL of 48% HF and 8 mL of 12M HCl mixed acid to prepare the typical “accordion” layered structure (Figure 2b,c). SEM characterization further confirms (Figure 2d–f) that the mixed-acid etched samples show a more significant interlayer expansion effect and regular two-dimensional flake structure, providing optimized diffusion channels for ion transport. Wu et al. [51] used the mixed-acid etching method to prepare few-layer or single-layer V-based MXene, increasing active sites, and combined it with carbon nanotube (CNT) composite technology to build a two-dimensional/one-dimensional V_2_CT*_x_*/CNT electrode. In this electrode, the three-dimensional network of CNTs suppresses the aggregation between MXene layers, reducing the charge transfer resistance to 32% of pure MXene and increasing the surface capacitance of the composite electrode to 246.88 mF cm^−2^ (0.5 mA cm^−2^). These studies indicate that mixed-acid etching optimizes the interlayer structure, exposure of active sites, and charge transfer dynamics of MXene, providing an essential direction for developing high-energy storage performance MXene-based electrodes.

### 3.2. Hydrothermal Etching Method

**Figure 3:**
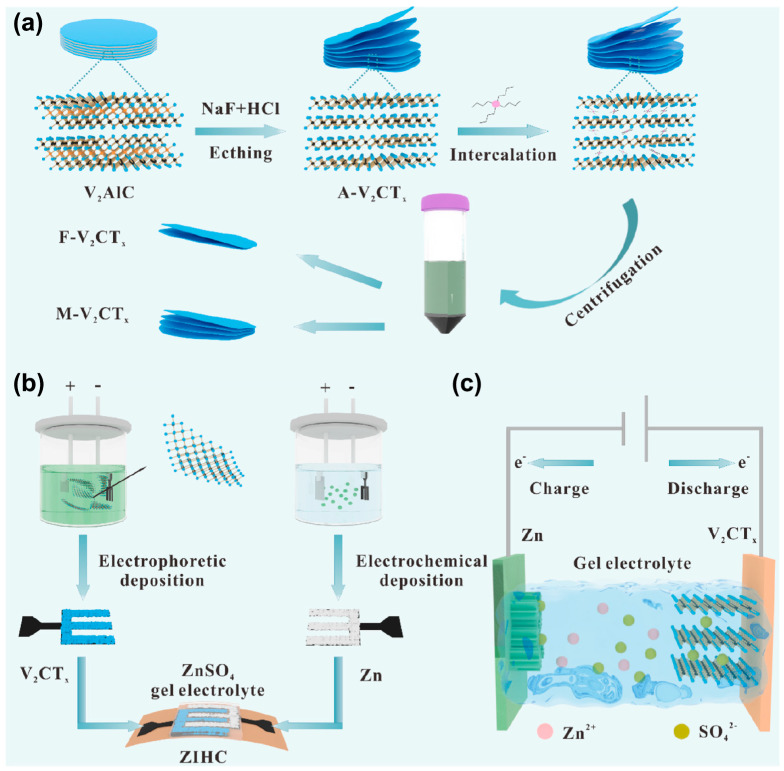
The fabrication process and mechanism of ZIHC based on V_2_CT*_x_*. (**a**) Controllable synthesis of layered V_2_CT*_x_* nanosheets. (**b**) Assembly strategy of flexible ZIHC devices. (**c**) The synergistic energy storage mechanism of ZIHC [50]. Copyright 2022, Elsevier.

Although the hydrofluoric acid etching method can effectively generate MXene materials, its high corrosiveness, safety risks, and environmental impact make most current research use hydrofluoric-acid-free preparations. Researchers have developed a new method for synthesizing Ti_3_C_2_ MXene, using a mixture of lithium fluoride (LiF) and hydrochloric acid (HCl) as an in situ-generated HF etching agent [56]. The advantage of this method is that Li^+^ ions can be embedded between the Ti_3_C_2_ layers during the etching process, increasing the interlayer distance, thus improving the ion diffusion rate and electrochemical specific capacity. The research team of Guo et al. [57] further explored other combinations of fluoride salts and hydrochloric acid as etching agents. Yet, results showed that ammonium fluoride (NH4F) and hydrochloric acid (HCl) required a reaction at 140 °C for 24 h in a Teflon-lined stainless steel autoclave to obtain pure MXene. Different MXene materials have different etching temperatures and selections for fluoride salts. Liu et al. [58] synthesized high-purity V_2_C MXene by etching V_2_AlC with sodium fluoride and hydrochloric acid at 90 °C for 72 h for lithium-ion batteries. Zhao et al. [50] etched multilayer/few-layer V_2_CT*_x_* MXene by the hydrothermal method, and the process is shown in Figure 3a. A two-dimensional V_2_CT*_x_* MXene material with an interlayer expanded accordion morphology was successfully prepared by selective chemical etching of the Al atom layer in the V_2_AlC precursor using a mixed solution of hydrochloric acid (HCl) and sodium fluoride (NaF). Subsequently, the V_2_CT*_x_* nanosheets were uniformly assembled on the surface of a flexible conductive substrate using the electrophoretic deposition (EPD) process to construct a high-performance flexible electrode (Figure 3b). Furthermore, a Zinc-Ion Hybrid Capacitor (ZIHC) was designed and assembled based on the electrode. Its energy storage mechanism (Figure 3c) can be analyzed as follows: During charging and discharging, zinc ions (Zn^2+^) diffuse through the electrolyte to the interlayer of V_2_CT*_x_* and reversibly react with the surface functional groups (such as –O, –F, etc.) in a pseudocapacitive reaction; at the same time, anions in the electrolyte (such as SO_4_^2−^) form a double-layer adsorption at the electrode/electrolyte interface, thus achieving efficient energy storage and release. This synergistic mechanism combines the fast kinetic characteristics of double-layer capacitance with the high energy density advantages of pseudocapacitance.

**Figure 4:**
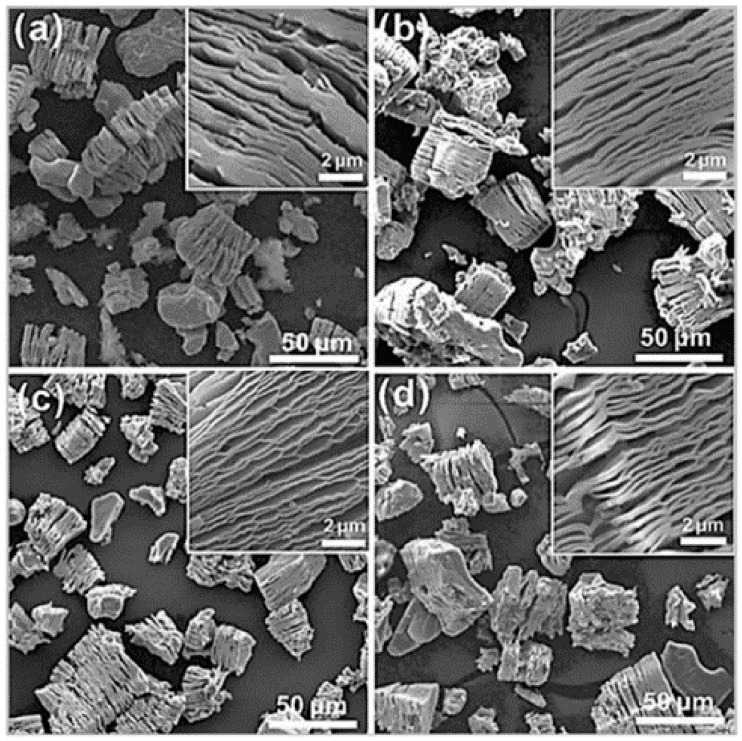
SEM images of V_2_AlC etched with LiF and HCl at 90 °C for (**a**) 72 h, (**b**) 96 h, (**c**) 120 h, and (**d**) 144 h V_2_C MXene. The insets show a high-magnification SEM [59]. Copyright 2020, IOP Science.

The traditional sodium fluoride/hydrochloric acid system struggles to achieve single-layer exfoliation of V_2_CT*_x_* MXene, a technical bottleneck that was broken by the team led by Guan et al. [59]. Research shows that the etching time significantly regulates the layered structure by using a LiF/HCl mixed solution to gradient temporally hydrothermally etch the V-based MXene precursor (90 °C, 72~144 h). SEM analysis indicates that as the etching time is extended from 72 h to 144 h, (Figure 4) the selective etching efficiency of the Al atomic layer improves, and the interlayer spacing gradually expands. Short etching times (72 h) tend to cause localized holes and non-uniform exfoliation defects, while gradually increasing the etching time results in the material forming an “accordion” like structure. However, at 144 h, collapse in the structure is evident, but the interlayer interface is stable at 120 h. This result reveals that extending the etching time can promote the diffusion of the etching agent and orderly deslipping of the Al layer, thereby enhancing the structural regularity and potential energy storage applications of the two-dimensional nanosheets. Thus, the two-dimensional nanosheets’ structural regularity and potential energy storage applications are enhanced. The preparation scheme of the V-MXenes is shown in Table 1.

The possible reactions in the synthesis process of V_2_CT*_x_* MXene are as follows:

$$V_{2}\mathrm{AlC}+3\mathrm{LiF}+3\mathrm{HCl}\to V_{2}{C+\mathrm{AlF}}_{3}+3\mathrm{LiCl}+1{.5H}_{2}↑$$

## 4. Application of V-MXene in Supercapacitors

**Figure 5:**
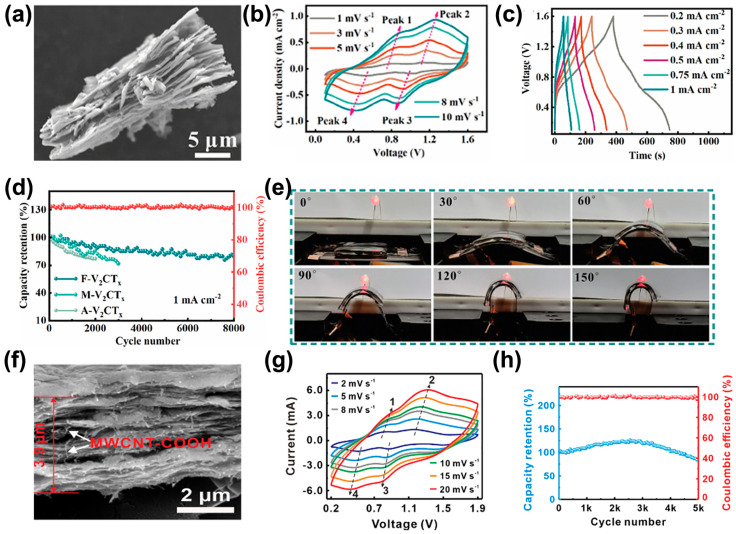
(**a**) SEM of V_2_CT*_x_*. (**b**) CV curves of ZIHC at 1 to 10 mV s^−1^ scan rate. (**c**) GCD curves of ZIHC at various current densities (0.2~1 mA cm^−2^). (**d**) Long-term cycling performance of A-ZIHC, M-ZIHC and F-ZIHC at 1 mA cm^−2^. (**e**) Digital images of the F-ZIHC powering an LED lamp under different bending states [50]. Copyright 2023, Elsevier. (**f**) SEM of V_2_CT*_x_*/CNT cathode. (**g**) CV curves of V/C-ZIMSC at 2.0~20.0 mV s^−1^ scan rate. (**h**) Cycling stability of V/C-ZIMSC at 3.2 mA cm^−2^ [51]. Copyright 2024, Elsevier.

Vanadium-based MXene (V-MXene) has shown broad application prospects in various fields due to its unique structure and excellent physicochemical properties, especially in energy storage, catalysis, electrochemical sensing, and environmental protection. Energy storage is mainly used in supercapacitor electrode materials, lithium-ion batteries, and sodium-ion batteries. The capacity decay of V-based MXene during cycling can be principally attributed to the material’s structural collapse and volume expansion. During the electrochemical reaction process, accompanied by the ion insertion/extraction, the volume of the electrode material will change. If the structure collapses, the interlayer distance of V-based MXene will become smaller, affecting the ion insertion/extraction kinetic rate [58]. At the same time, the number of active sites will decrease. Secondly, the surface functional groups of V-based MXene may have side reactions with the electrolyte, affecting the material performance, and there may be irreversible phase changes during cycling, leading to capacity decay [59]. Currently, researchers mainly alleviate the volume expansion through structural strategy engineering to construct layered heterostructures and form solid solution structures, as well as enhance the electronic conductivity of the host material (V-based MXene) through surface modification, such as composites with carbon materials, to suppress the capacity decay during cycling. Zhao et al. [50] constructed a few-layer V_2_CT*_x_*-based flexible zinc ion hybrid capacitor (ZIHC) through electrophoretic deposition technology. Its unique two-dimensional exfoliated structure exposed a high density of surface-active sites (Figure 5a) and shortened the diffusion path of Zn^2+^, enabling the device to achieve a capacitance of 54.12 mF cm^−2^ under 2M ZnSO_4,_ 0.1 mA cm^−2^. After 8000 cycles, the capacity retention rate was 81.48% (Figure 5b–d). The material maintained stable electrochemical performance under extreme mechanical deformation (bending angle 0~150°, multi-axis bending load cycle), confirming its intrinsic flexible characteristics and structural tolerance (Figure 5e). This excellent mechanical–electrochemical coupling stability broke through the performance bottleneck of traditional energy storage materials under dynamic service environments, providing a key material basis for the development of flexible/stretchable energy devices that can withstand complex deformations (such as implantable medical devices, bright fabric integrated systems), highlighting the strategic value of MXene-based materials in the next generation of wearable electronics. Wu et al. [51] further broke through performance limits through a heterogeneous composite strategy: using low-temperature (0~5 °C) gradient etching combined with carbon nanotube (CNT) composite technology, they constructed a two-dimensional/one-dimensional V_2_CT*_x_*/CNT electrode. In this, the three-dimensional network of CNTs inhibited the interlayer agglomeration of MXene (Figure 5f), reducing the charge transfer resistance to 32% of pure MXene and synergizing the pseudocapacitive active sites of MXene, increasing the composite electrode surface capacitance to 246.88 mF cm^−2^ (0.5 mA cm^−2^). After 10,000 cycles (3.2 mA cm^−2^), the capacity retention rate was >90% (Figure 5h).

**Figure 6:**
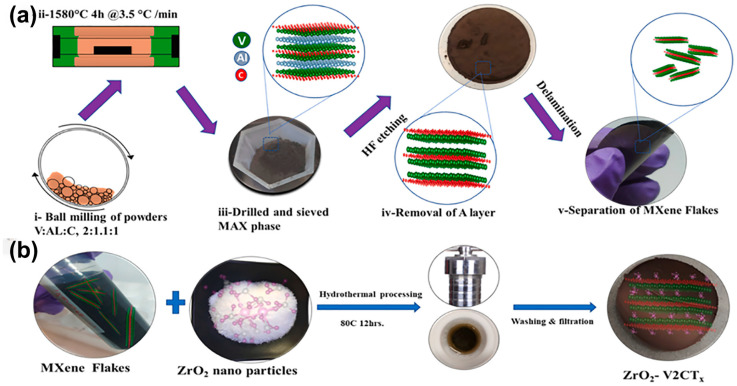
(**a**) Synthesis of MAX and MXene flakes. (**b**) Schematic illustration for the synthesis of ZrO_2_-V_2_CT*_x_* composites [61]. Copyright 2022, Elsevier.

V_2_CT*_x_* has applications not only in ZnSO_4_ electrolyte. Majid et al. [60] used Ti_3_C_2_T*_x_* and V_2_CT*_x_* colloidal solutions to induce self-assembly through a saturated sodium chloride solution. The fabricated free-standing and binder-free MXene heterostructure film showed characteristic redox peaks, which led to more stable capacitance across the entire testing range. The modified MXene structure has a high volumetric capacity of about 1473 F cm^−3^ in 3M H_2_SO_4_ electrolyte, with a maximum volumetric power and energy density of 0.03 W cm^−3^ and 12.3 Wh cm^−3^, respectively, and no capacity decay after 50,000 cycles. The self-assembly of two 2D materials has a stable structural state, and Rizwan et al. [61] prepared V_2_CT*_x_* by etching with hydrofluoric acid (HF) as mentioned above. They also prepared ZrO_2_-V_2_CT*_x_* via a hydrothermal method at 80 °C for 12 h. Introducing zirconium oxide reduces the electrostatic attraction between V_2_CT*_x_* flakes, but the overall structure remains unchanged (Figure 6). In ZrO_2_-V_2_CT*_x_*, zirconium provides accessible diffusion sites and accelerates diffusion dynamics to drive pseudocapacitive behavior, exhibiting 447 F g^−1^ at a scan rate of 5 mV s^−1^ in 3M H_2_SO_4_ electrolyte using plastic Swagelok test cells. Moreover, under 1 A g^−1^ testing for 10,000 charge/discharge tests, the Coulombic efficiency is close to 100%, indicating that the electrode durability and stability significantly improved after the self-assembly of zirconium oxide and V_2_CT*_x_*. This represents another breakthrough in the electrochemical stability of V-based MXene materials.

**Figure 7:**
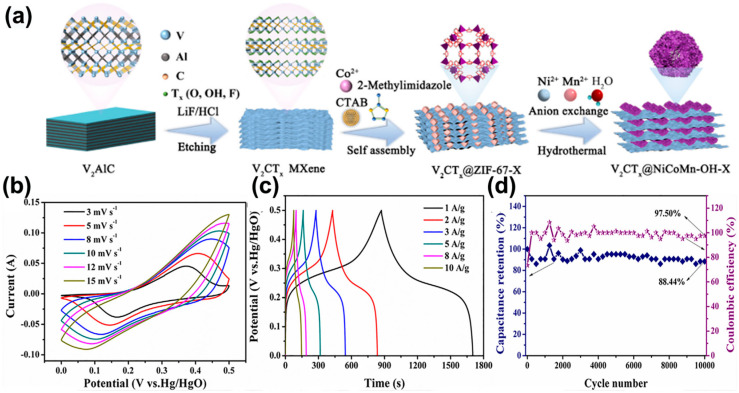
(**a**) Schematic illustration for the synthesis of V_2_CT*_x_*@NiCoMn-OH composites. (**b**) CV curves at different scan rates in 3M KOH electrolyte. (**c**) GCD curves at various current densities and (**d**) cycling stability and Coulombic efficiency at 20 A g^−1^ for 10,000 cycles [63]. Copyright 2023, Elsevier.

V_2_CT*_x_* materials exhibit excellent energy storage effects in acidic electrolytes, and some researchers have also studied them in alkaline electrolytes. Yadav et al. [62] innovatively developed a metal–organic framework (MOF)-assisted synthesis strategy, successfully preparing a vanadium carbide composite material (V_2_CT*_x_*@C) with a three-dimensional carbon network confinement structure. Through the topological structure reconstruction induced by the MOFs template, the initially stacked V_2_CT*_x_* MXene nanosheets were dissociated and reassembled into a nano-disk array rich in carbon matrix, achieving optimization of three key structures: specific surface area, electronic conductivity, and effective contact area of the electrode. Yu’s team [63] successfully constructed a 2D@3D V_2_CT*_x_*@NiCoMn-OH-20 nanocomposite with a hierarchical heterostructure, which innovatively integrates the dual-function characteristics of supercapacitors and electrochemical sensors. The ZIF-67 precursor was uniformly anchored on the surface of V_2_CT*_x_* nanosheets through an electrostatic self-assembly strategy, and the active component distribution environment was accurately controlled by combining anion exchange reactions to achieve molecular-level coupling of the three-dimensional porous framework with the two-dimensional layered matrix. Structural characterization confirmed that this heterostructure effectively suppressed the interlayer stacking of the two-dimensional nanosheets, increasing the specific surface area to 385 m^2^ g^−1^, and constructed continuous ion transport channels. The optimized V_2_CT*_x_*@NiCoMn-OH-20 electrode exhibited a high specific capacity of 827.45 C g^−1^ at a current density of 1 A g^−1^, a 2.7-fold increase compared to a single component and a capacity retention rate of 88.44% after 10,000 cycles (Figure 7), which is attributed to the buffering effect of V-O-Co bond cooperation on structural stress at the heterogeneous interface. The assembled symmetrical supercapacitor achieved an energy density of 88.35 Wh kg^−1^ and a power density of 750 W kg^−1^.

**Figure 8:**
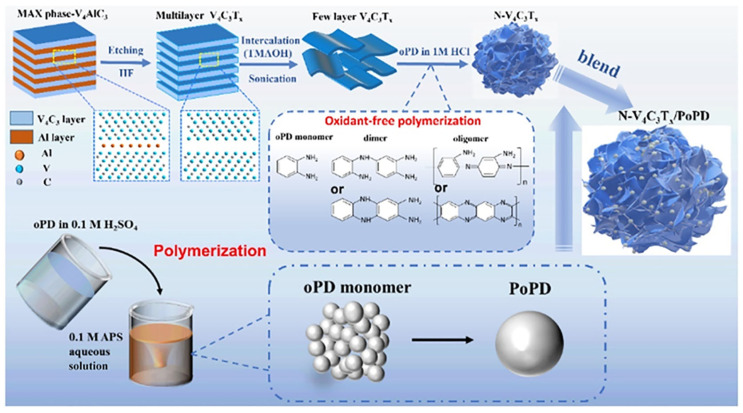
Schematic diagram of the preparation of V_4_C_3_T*_x_* and the preparation of N-V_4_C_3_T*_x_*/PoPD mixture [67]. Copyright 2023, ACS Publications.

**Figure 9:**
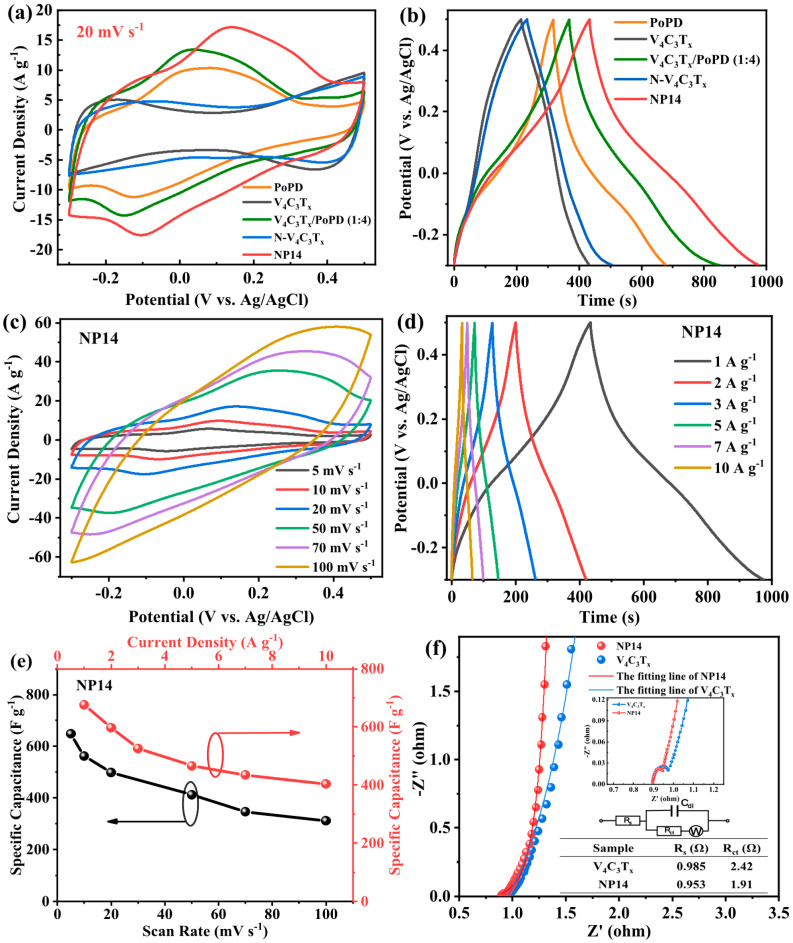
(**a**) CV curves of V_4_C_3_T*_x_*, N-V_4_C_3_T*_x_*, PoPD, V_4_C_3_T*_x_*/PoPD (1:4), and NP14 electrodes in 1 M H_2_SO_4_ electrolyte. (**b**) GCD curves of V_4_C_3_T*_x_*, N-V_4_C_3_T*_x_*, PoPD, N-V_4_C_3_T*_x_*/PoPD (1:4), and NP14 electrodes at 1 A g^−1^. (**c**) Specific capacitance of the NP14 electrode at different scan rates. (**d**) GCD curves of the NP14 electrode at different current densities. (**e**) Plots of specific capacitance versus scan rate and current density of the NP14 electrode. (**f**) Nyquist plots of V_4_C_3_T_x_ and NP14 electrodes [67]. Copyright 2023, ACS Publications.

Based on the study of the structural characteristics and energy storage mechanisms of V_2_CT*_x_* MXene, we further focused on the research of higher-order homologous material V_4_C_3_T*_x_*, exploring the effects of the complete addition of transition metal layers on its specific surface area, conductivity, and electrochemical performance. Wang et al. [65] successfully prepared two-dimensional multilayer V_4_C_3_ MXene material by selectively removing Al layers from V_4_AlC_3_ precursor through a topological chemical etching strategy, achieving a specific capacitance of 209 F g^−1^ at a scan rate of 2 mV s^−1^. Subsequently, Cheng et al. [67] addressed the issues of easy oxidation of the MXene surface and the stacking limitations of its layered structure by adopting poly-o-phenylenediamine (PoPD) modified vanadium carbide MXene (N-V_4_C_3_T*_x_*). Using an in situ oxidant-free polymerization and redox synergistic process (the preparation process is shown in Figure 8), PoPD nanoparticles were uniformly anchored onto the surface of N-V_4_C_3_T*_x_* nanosheets, forming a three-dimensional conductive network composite system. Through proportional optimization, it was found that the optimal ratio of N-V_4_C_3_T*_x_* and PoPD powder is 1:4 (abbreviated as NP14). The strong chemical bonding between the amino group (–NH_2_) and the –O/–F groups on the MXene surface inhibits the stacking of nanosheets, constructing continuous electron transport channels and increasing the electrical conductivity of the material. The composite electrode achieved a specific capacitance of 676.5 F g^−1^ at a current density of 1 A g^−1^, which is 2.48 times higher than that of the pure V_4_C_3_T*_x_* electrode, and after 10,000 cycles, it still maintained a capacity retention rate of 95%. This is attributed to the synergistic effect of the π-π conjugated system of PoPD and the nitrogen-containing functional groups on the MXene surface, reducing the charge transfer resistance by 68%. As a negative electrode material, N-V_4_C_3_T*_x_* exhibited a high reversible capacity of 561.2 mAh g^−1^ at 0.1 A g^−1^ and still maintained 382.4 mAh g^−1^ at a high rate of 2 A g^−1^. Its performance advantage stems from the synergistic effect of the pseudocapacitance contribution induced by N doping and the interlayer expansion effect of MXene (Figure 9). Through a molecular interface design strategy, this work overcame the dual bottlenecks of limited capacity and insufficient cycle stability of MXene materials, providing a new paradigm for developing high-cost performance energy storage devices.

**Figure 10:**
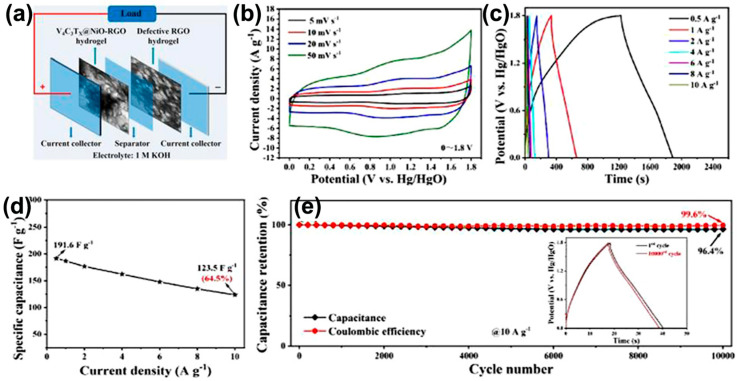
(**a**) The schematic illustration of the construction of the ASC device. (**b**) CV curves of the ASC at different scan rates in the voltage window of 0~1.8 V. (**c**) GCD curves of the ASC at different scan rates in the voltage window of 0~1.8 V. (**d**) Specific capacitance calculated from GCD curves at various current densities. (**e**) Cyclability and Coulombic efficiency at 10 A g^−1^ [68]. Copyright 2023, Royal society of chemistry.

The two-dimensional, multi-layered V_4_C_3_ MXene material is not limited to acidic electrolytes but exhibits good energy storage performance in alkaline KOH electrolytes. Table 2 outlines the applications of V-MXenes and modified supercapacitor electrode materials. Researchers [68,69,70,71,72,73,74,75,76] developed a high-performance V_4_C_3_T*_x_*@NiO-RGO heterostructure hydrogel and a defect-engineered DRGO hydrogel through a multi-step synergistic assembly strategy. They integrated them to create a high-energy density asymmetric supercapacitor (ASC). Using the hydrothermal method, NiO nanoplates with high pseudocapacitance characteristics were uniformly deposited on the surface of highly conductive V_4_C_3_T*_x_* to form a face-to-face heterogeneous interface with strong chemical bonding. Combined with the graphene oxide self-assembly strategy, a three-dimensional interconnected network was constructed, effectively inhibiting the stacking of nanoplates. As a result, the material exhibited an ultra-high specific capacitance of 191.6 F g^−1^ at 0.5 A g^−1^ and 123.5 F g^−1^ at 10 A g^−1^ (Figure 10).

## 5. Conclusions and Prospects

### 5.1. Conclusions

This paper reviews the characteristics, preparation methods, and applications of vanadium-based MXene in the field of supercapacitors. V-MXene, with its multivalent state characteristics, high conductivity, large specific surface area, and adjustable interlayer spacing, demonstrates great potential in the energy storage field. Through different etching methods and surface functionalization, researchers can effectively enhance the electrochemical performance of V-based MXene, making its application prospects broad in areas such as supercapacitors and batteries. However, the preparation and application of V-based MXene still face many challenges, such as the generation of by-products during the etching process, poor cyclic stability of the material, and optimization of electrochemical performance. In practice, it still needs to be clarified how the structure of the MXenes, the chemical functional groups on the surface, and the long-term stability in various applications can be better controlled. Moreover, research based on vanadium-based MXene composite materials and heterostructures will also be an important direction for future development. Through synergistic effects with other functional materials, there is hope to enhance its application in energy storage further.

### 5.2. Prospects

In conclusion, V-based MXenes hold significant promise for supercapacitor applications due to their unique structure and favorable surface functionality. However, several challenges remain. With careful structural design and surface modification, we believe these obstacles can be overcome, expanding the use of V-based MXenes beyond supercapacitor electrodes. Future research should focus on the following directions:

1. Enhancing synthesis efficiency and purity: Developing more efficient and green synthesis processes, reducing by-product generation, and optimizing reaction conditions to obtain high-purity V-based MXene.
2. Structure and function optimization: Explore different surface modification and functionalization strategies to enhance the stability, conductivity, and reactivity of the material, further improving its electrochemical performance.
3. Composite materials and heterostructures: By compounding with other two-dimensional materials or nanomaterials, novel heterostructures are developed. Utilizing the built-in electric field within the heterojunction can accelerate electron transfer and expose more electrochemical sites at the grain boundaries, thereby synergistically enhancing the electrochemical performance.
4. Large-scale production and practical applications: advancing the fabrication technologies of V-based MXene materials at industrial scales, paving the way for their energy storage and conversion applications.

## Figures and Tables

**Table 1:** Summary of the preparation schemes of V-MXenes.

Material	Etching Method	Synthesis Time	Synthesis Temperature	Capacity	Reference
V_2_CT*_x_*	NaF + HCL (6 M)	7 day	90 °C	0.1 mA cm^−2^ 54.12 mF cm^−2^	[50]
V_2_CT*_x_*	LiF + HCL	120 h	90 °C	2 mV s^−1^ 164 F g^−1^	[59]
V_2_CT*_x_*	LiF + HCL (6 M)	24 h	35 °C	2 mV s^−1^ 1473 F cm^−3^	[60]
V_2_CT*_x_*	50% HF	92 h	25 °C	5 mV s^−1^ 1200 F g^−1^	[61]
V_2_CT*_x_*	48% HF	120 h	70 °C	2 mV s^−1^ 1842 F g^−1^	[62]
V_2_CT*_x_*	LiF + HCL (12 M)	72h	90 °C	1 A g^−1^ 827.45 C g^−1^	[63]
V_2_CT*_x_*	LiF + HCL (12 M)	72 h	90 °C	1 A g^−1^ 1658.2 F g^−1^	[64]
V_4_C_3_T*_x_*	40% HF	3 day	25 °C	5 mV s^−1^ 330 F g^−1^	[65]
V_4_C_3_T*_x_*	40% HF	96 h	55 °C	2 mV s^−1^ 209 F g^−1^	[66]

**Table 2:** Review of the application of V-MXenes and modified supercapacitor electrode materials.

Electrode Material	Electrolyte	Potential Window	Capacitance	Scan Rate/Current Density	Reference
V_2_CT*_x_*	2 M Zn_2_SO_4_	0.1~1.6	54.12 mF cm^−2^	0.1 mA cm^−2^	[50]
V_2_CT*_x_*/CNT	2 M Zn_2_SO_4_	0.2~1.9	246.88 mF cm^−2^	0.5 mA cm^−2^	[51]
V_2_C	1 M Na_2_SO_4_	−0.3~−0.9	164 F g^−1^	2 mV s^−1^	[59]
V_2_CT*_x_*	3 M H_2_SO_4_	−0.4~0.2	1473 F cm^−3^	2 mV s^−1^	[60]
V_2_CT*_x_*/ZrO_2_	3 M H_2_SO_4_	−0.5~0.3	1200 F g^−1^	5 mV s^−1^	[61]
V_2_CT*_x_*@C	1 M H_2_SO_4_	0.0~1.2	551 F g^−1^	2 A g^−1^	[62]
V_2_CT*_x_*@NiCoMn-OH	3 M KOH	0.0~0.5	827.45 C g^−1^	1 A g^−1^	[63]
V_2_CT*_x_*/Co-LDH	6 M KOH	−0.1~0.4	1005 F g^−1^	1 A g^−1^	[64]
V_4_C_3_T*_x_*	1 M H_2_SO_4_	−0.4~0.4	330 F g^−1^	5 mV s^−1^	[65]
V_4_C_3_	1 M H_2_SO_4_	−0.3~0.1	209 F g^−1^	2 mV s^−1^	[66]
V_4_C_3_T*_x_*/POPD	1 M H_2_SO_4_	−0.2~0.4	676.5 F g^−1^	1 A g^−1^	[67]
V_4_C_3_T*_x_*@NiO-RGO	1 M KOH	−1.0~0.8	1021 F g^−1^	0.5 A g^−1^	[68]
V_2_CT*_x_*/Ti_3_C_2_T*_x_*	1 M H_2_SO_4_	−0.6~0.2	365 F g^−1^	1 A g^−1^	[69]
V_2_C	1 M Na_2_SO_4_	−1.0~−0.2	120 F g^−1^	2 mV s^−1^	[70]
V_2_CT*_x_*	2 M Zn_2_SO_4_	−0.9~0.3	481 F g^−1^	1 A g^−1^	[71]
V_2_CT*_x_*@Si	1 M Zn_2_SO_4_	0~1.0	557 F g^−1^	1 A g^−1^	[72]
V_2_CT*_x_*	1 M LiOH	−1.4~0.8	386 F g^−1^	2 mV s^−1^	[73]
V_2_CT*_x_*/NiV-LDH	6 M KOH	0.0~0.5	1658.2 F g^−1^	1 A g^−1^	[74]
V_4_C_3_T*_x_*	1 M H_2_SO_4_	−0.3~0.3	210 F g^−1^	10 mV s^−1^	[75]
NiCoAl-LDH/V_4_C_3_T*_x_*	1 M KOH	−0.1~0.5	627 F g^−1^	1 A g^−1^	[76]

## Data Availability

No new data were created or analyzed in this study.
